# *Helicobacter pylori* eradication affects platelet count recovery in immune thrombocytopenia

**DOI:** 10.1038/s41598-020-66460-5

**Published:** 2020-06-10

**Authors:** Ayoung Lee, Junshik Hong, Hyunsoo Chung, Youngil Koh, Soo-Jeong Cho, Ja Min Byun, Sang Gyun Kim, Inho Kim

**Affiliations:** 1 Division of Gastroenterology, Department of Internal Medicine and Liver Research Institute, Seoul National University College of Medicine, Seoul, Korea; 2 Division of Hematology and Medical Oncology, Department of Internal Medicine, Seoul National University Hospital, Cancer Research Institute, Seoul National University College of Medicine, Seoul, Korea

**Keywords:** Haematological diseases, Gastroenterology

## Abstract

*Helicobacter pylori (H. pylori)* infection is on the rise as a cause of immune thrombocytopenia (ITP). It has been suggested that platelet recovery can be achieved following successful microbial eradication, although, the exact pathophysiology has yet to be fully elucidated. This study evaluated the long-term effects of *H. pylori* eradication monotherapy on platelet count recovery in patients with ITP. *H. pylori* eradication was analysed in 61 ITP patients. Patients who maintained a complete response (CR) for more than six months were classified as sustained responders (SR). The prevalence of *H. pylori* infection was 54.3% (75/138), and the success rate of eradication with first-line therapy was 71.4% (35/49). Patients who had achieved a CR at 2 months maintained a higher platelet count thereafter. At 1 year following eradication, platelet counts had increased 2.78 times in the eradicated group, 1.36 times in the sustained infection group, and 1.33 times in the no infection group compared with the baseline (*P* = 0.016).

## Introduction

Immune thrombocytopenia (ITP) causes a reduction in platelet counts to 100 × 10^9^/L or less due to acquired blood abnormalities such as autoantibodies or immunocomplexes; however, the pathophysiology is yet to be elucidated^1,2^. The pathophysiology of this disorder has revealed a potential link to *Helicobacter pylori (H. pylori)* as one of the aetiologies. *H. pylori* is associated with gastritis, peptic ulcers, gastric adenocarcinoma, and MALToma. In addition, some studies have reported an association between *H. pylori* and extragastrointestinal diseases including haematological diseases (ITP and unexplained iron deficiency anemia), cardiovascular diseases (ischaemic heart diseases), neurological disorders (stroke, Parkinson’s disease, Alzheimer’s disease), obesity, and skin disorders^3–5^.

Based on recent systematic reviews, more than half of patients have successfully recovered platelet counts following *H. pylori* eradication treatment, and the clinical course was most pronounced in geographic regions with high baseline *H. pylori* infection rates^6,7^. Based on these findings, the Maastricht V guidelines strongly recommend the treatment of *H. pylori* eradication for chronic ITP; likewise, the Korean Helicobacter Treatment Guidelines, revised in 2013, also recommend treatment of *H. pylori* in ITP patients^8,9^. However, due to variances in prevalence by population for *Helicobacter* infections, conflicting results have caused the eradication of *H. pylori* in ITP patients to be controversial.

Current guidelines recommend corticosteroids and gamma globulins as the first-line treatments for ITP^1,10^. However, many patients are dependent on corticosteroids, and the efficacy of intravenous gamma globulins (IVIG) is transient. Moreover, some patients become unresponsive to subsequent treatment, which is a concerning adverse effect associated with immunosuppressants. The eradication of *H. pylori* infection possibly affects the course of ITP thus inducing remission and could be a long-term treatment strategy. Currently, there are few large-scale prospective studies designed for the degree of platelet recovery based on the results of *H. pylori* eradication in ITP patients, therefore, the timing of the treatment response has yet to be established as seen in steroid or IVIG treatments^11^. To our knowledge, there are no large-scale prospective studies that have seen the long-term effects on platelet response following *H. pylori* eradication. In previous retrospective studies, other treatments are often performed together with eradication therapy, making it difficult to examine the eradication effect alone. The aim of this study was to clarify the long-term effect of *H. pylori* eradication monotherapy on the platelet count recovery in patients with ITP.

## Results

### Eradication rate and relationship with duration of therapy

The prevalence of *H. pylori* infection was 54.3% (75/138) in our study population. We calculated the eradication rate with first-line treatment; among the 75 patients diagnosed as *H. pylori* infection, 49 patients were treated with first-line regimen for eradication. The success rate of eradication was 71.4% (35/49). Twelve of the 14 patients who had failed the eradication with first-line treatment received a second-line treatment, where in 11 of them, eradication was achieved. The overall eradication with first and second therapy was 93.9% (46/49).

For 49 patients who performed eradication with first-line therapy, we analysed the association between the success rate of eradication and the duration of treatment (Supplement Table 1). The success rate of eradication with first-line therapy tended to increase with long treatment duration, but there was no statistical significance (66.7% vs. 90.0%, *P* = 0.244).

### Baseline characteristics of final enrollment

Detailed analyses were conducted for each of the 61 patients, who were either *H. pylori*-positive and without any concomitant therapy within 2 months following the eradication, or *H. pylori*-negative and received no treatment for 2 months from the time of diagnosis. The baseline characteristics of 61 patients that were assessed for platelet response at 2 months without concomitant therapy within 2 months follow-up were shown in Table 1. Furthermore, no statistical differences in age, sex distribution, baseline platelet count, mean platelet volume, haemoglobin, reticulocyte, neutrophil- lymphocyte count ratio, antinuclear antibody positivity, CRP or ESR were found between the three groups. The baseline white blood cell count and absolute neutrophil count were significantly higher in the eradicated group compared to the other two groups (*P* < 0.05, respectively).

**Table 1:** Baseline Characteristics of the study population between eradicated group, sustained infection group, no infection group.

	Eradicated group (n = 26)		Sustained infection group (n = 10)		No infection group (n = 25)		P-value
	N	Mean ± SD	N	Mean ± SD	N	Mean ± SD	
Age (year)	26	52.5 ± 12.3	10	56.0 ± 16.6	25	48.9 ± 20.2	0.490^†^
Sex (male)	8	30.8%	4	40.0%	9	36.0%	0.853^*^
Platelet, × 10^9^/L [range]	26	50.2 ± 23.5 [8–89]	10	63.3 ± 33.9 [21–98]	25	60.6 ± 23.4 [27–87]	0.238^†^
MPV, fL	22	10.9 ± 2.1	7	11.8 ± 1.2	24	11.2 ± 2.7	0.680^†^
WBC, × 10^3^/uL	26	8.0 ± 2.7	10	5.7 ± 1.9	25	5.6 ± 2.0	0.002^†^
Neutrophil, × 10^3^/uL	26	5.2 ± 2.3	10	3.2 ± 1.4	25	3.0 ± 1.5	0.000^†^
Lymphocyte, × 10^3^/uL	26	2.2 ± 1.1	10	2.4 ± 0.9	25	1.8 ± 0.8	0.146^†^
Monocyte, × 10^3^/uL	26	0.4 ± 0.2	10	0.5 ± 0.2	25	0.5 ± 0.7	0.589^†^
Eosinophil, × 10^3^/uL	26	0.2 ± 0.4	10	0.1 ± 0.1	24	0.1 ± 0.1	0.554^†^
Basophil, × 10^3^/uL	26	0.0 ± 0.0	9	0.0 ± 0.0	24	0.0 ± 0.0	0.510^†^
Haemoglobin, g/dL	26	12.7 ± 2.7	10	13.2 ± 2.8	25	13.2 ± 1.7	0.760^†^
Reticulocyte, g/dL	15	72.6 ± 27.7	3	39.8 ± 6.4	14	62.4 ± 13.8	0.059^†^
NLR	26	2.8 ± 3.2	10	1.4 ± 0.5	25	1.8 ± 0.9	0.144^†^
Antinuclear antibody positivity	2	15.4% (2/13)	1	14.3% (1/7)	7	38.9% (7/18)	0.248^*^
CRP, mg/dL	13	0.8 ± 1.5	8	1.6 ± 3.3	17	0.6 ± 1.6	0.504^†^
ESR, mm/h	10	24.3 ± 25.9	8	22.8 ± 35.1	13	17.3 ± 15.2	0.781^†^

^*^Pearson Chi-square test.

^†^ANOVA.

N, number of patients; SD, standard deviation; MPV, Mean platelet volume; WBC, White blood cell; NLR, Neutrophil lymphocyte ratio; CRP, C-reactive protein; ESR, erythrocyte sedimentation rate.

### The response and trend of the platelet counts following *H. pylori* eradication

Platelet counts during the 5-year follow-up period for the eradicated, sustained infection, and no infection groups in 61 patients who had no concomitant therapy within the 2-month follow-up are depicted in Table 2 and Fig. 2. The trend changes in platelet recovery increased from baseline and were maintained during the follow-up period after successful eradication. However, they did not show significant differences in every period of assessment. The trend for the platelet counts in the sustained infection group, and the no infection group were similar.

**Table 2:** Comparison of platelet counts for the 5-year follow-up among groups with *H. pylori* infection status.

	Eradicated group		Sustained infection group		No infection group		P-value
	N	Mean ± SD	N	Mean ± SD	N	Mean ± SD	
Baseline	26	50.2 ± 23.5	10	63.3 ± 33.9	25	60.6 ± 23.4	0.238
At 2 months	26	96.5 ± 68.8	10	69.1 ± 34.1	25	73.8 ± 52.3	0.279
At 6 months	19	94.6 ± 71.7	6	74.3 ± 25.3	21	76.2 ± 29.5	0.477
At 1 years	17	110.2 ± 76.0	7	75.3 ± 55.7	20	68.0 ± 27.9	0.071
At 1.5 years	16	128.9 ± 90.2	3	71.3 ± 29.7	16	95.3 ± 71.5	0.349
At 2 years	16	98.9 ± 81.2	7	69.1 ± 25.7	14	76.1 ± 45.0	0.465
At 2.5 years	15	110.6 ± 97.0	4	78.8 ± 31.3	13	66.7 ± 32.9	0.272
At 3 years	13	100.8 ± 78.9	6	65.5 ± 21.7	8	53.9 ± 16.7	0.175
At 3.5 years	6	138.2 ± 112.4	3	63.7 ± 35.2	9	66.0 ± 34.1	0.155
At 4 years	9	107.9 ± 75.8	5	72.2 ± 46.6	9	75.3 ± 33.9	0.393
At 4.5 years	5	96.6 ± 84.3	2	19.5 ± 23.3	8	71.3 ± 45.6	0.342
At 5 years	5	92.4 ± 88.6	4	54.8 ± 41.8	5	47.6 ± 14.9	0.460

The units of mean and SD are marked with × 109/L.

*ANOVA test.

N; number of patients, SD; standard deviation.

**Figure 1:**
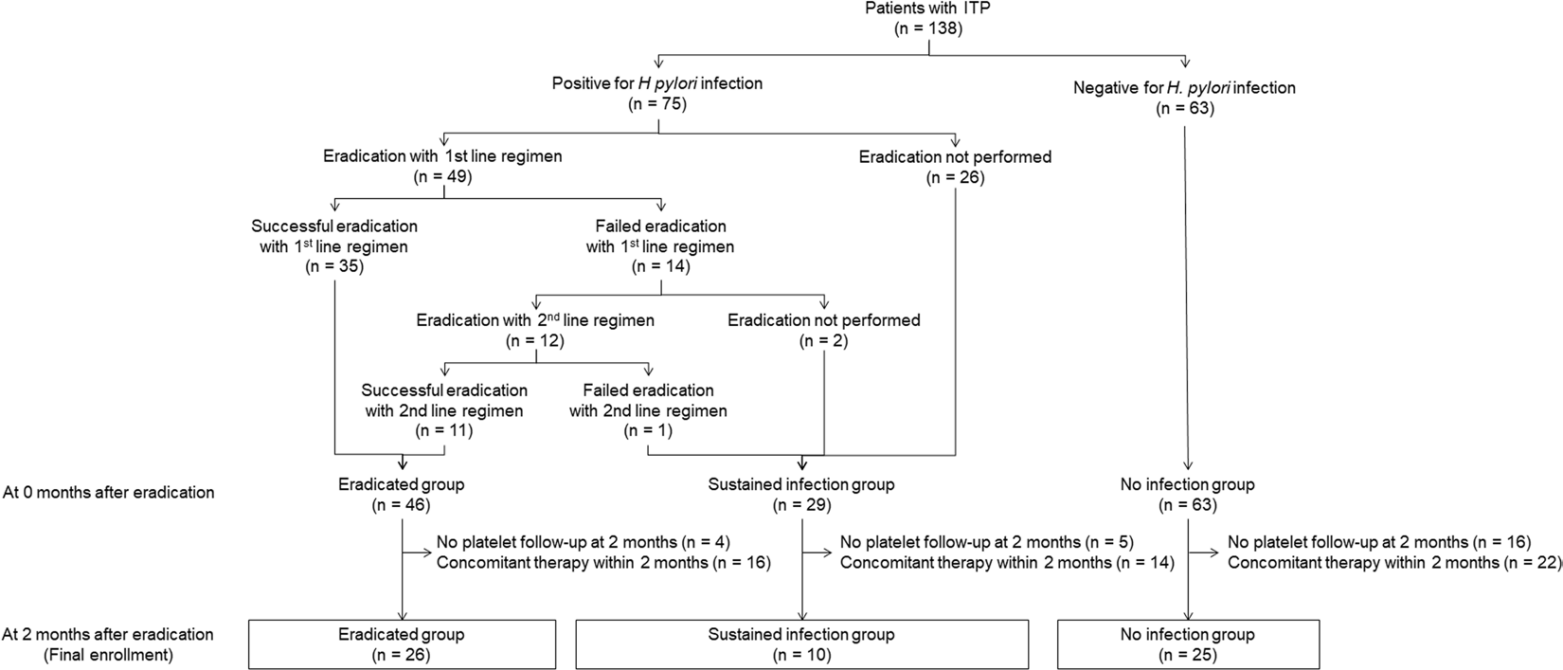
Patient Selection Flow Chart.

**Figure 2:**
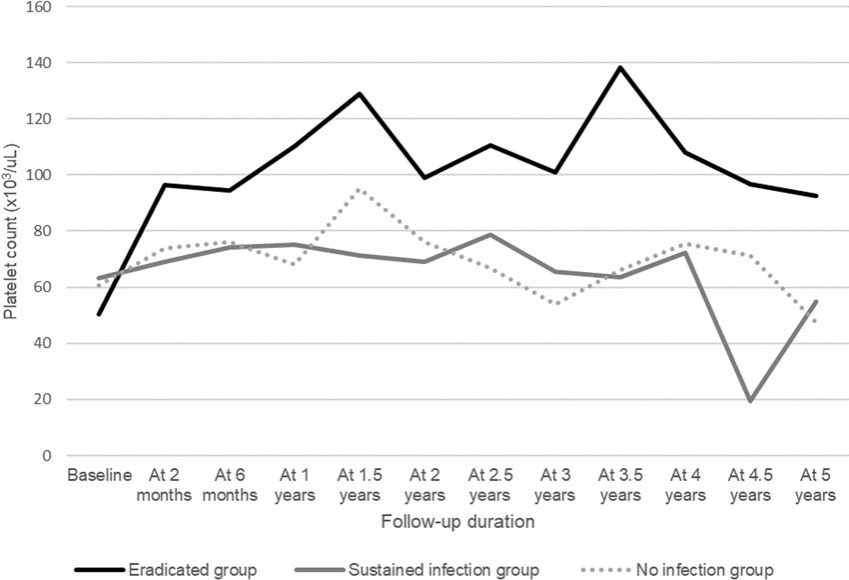
Trend of Platelet Counts during 5-year Follow-Up among Groups with *H. pylori* Infection Status.

**Figure 3:**
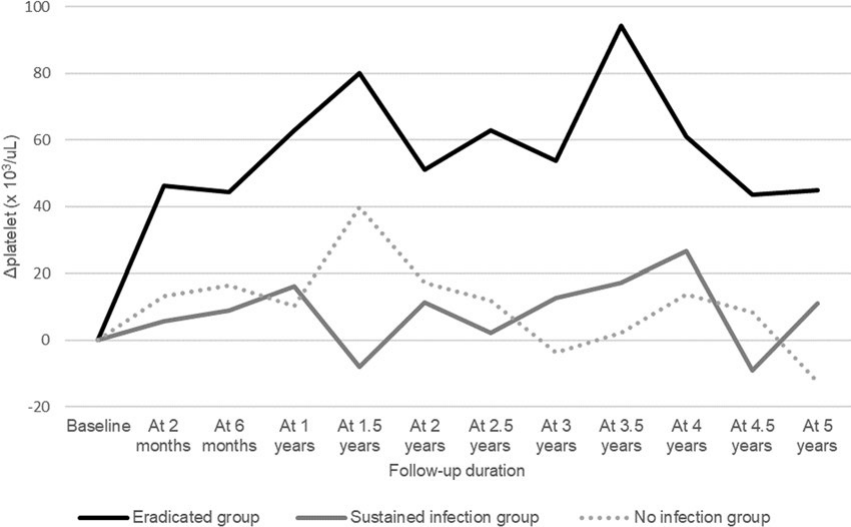
Trend of delta Platelet Counts during 5-year Follow-Up among Groups with *H. pylori* Infection Status.

The trends for differences in platelet counts are depicted in Table 3 and Fig. 3. The difference in platelet counts was defined as a delta platelet in this study and was calculated with their baseline at the time of assessment. One-year post infection, the eradicated groups’ platelets increased 62.7 ± 73.0, sustained infection group 16.0 ± 52.9, and no infection group 10.2 ± 32.3 compared to the baseline platelets (P = 0.016). During the observation period, platelets in the eradicated group retained a 1.89 to 2.87-fold increase compared to the baseline. The difference in delta platelet counts between the three groups was statistically significant only at the first year of follow-up, but it remained consistently higher than the baseline PLT in the eradicated group, although it was statistically insignificant.

**Table 3:** Comparison of delta platelet counts during 5-year follow-up among groups with *H. pylori* infection status.

	Eradicated group		Sustained infection group		No infection group		*P*-value	
	N	Mean ± SD	N	Mean ± SD	N	Mean ± SD		
At 2 months	26	46.2 ± 66.7 (2.33)	10	5.8 ± 24.9 (1.18)	25	13.3 ± 53.7 (1.41)	0.295^*^	0.062^†^
At 6 months	19	44.3 ± 66.1 (2.24)	6	9.0 ± 16.7 (1.36)	21	16.3 ± 29.4 (1.45)	0.215^*^	0.122^†^
At 1 years	17	62.7 ± 73.0 (2.78)	7	16.0 ± 52.9 (1.36)	20	10.2 ± 32.3 (1.33)	0.141^*^	**0.016**^**†**^
At 1.5 years	16	80.1 ± 84.6 (2.97)	3	−8.0 ± 8.5 (0.93)	16	39.9 ± 75.7 (2.05)	0.001^*^	0.136^†^
At 2 years	16	51.0 ± 71.1 (2.16)	7	11.4 ± 24.7 (1.42)	14	17.3 ± 52.1 (1.54)	0.062^*^	0.191^†^
At 2.5 years	15	62.9 ± 89.1 (2.30)	4	2.3 ± 27.2 (1.11)	13	11.8 ± 35.2 (1.33)	0.204^*^	0.092^†^
At 3 years	13	53.7 ± 74.9 (2.28)	6	12.7 ± 31.3 (1.52)	8	−3.8 ± 24.3 (1.04)	0.220^*^	0.079^†^
At 3.5 years	6	94.3 ± 114.5 (3.55)	3	17.3 ± 34.0 (1.52)	9	2.2 ± 32.1 (1.14)	0.305^*^	0.073^†^
At 4 years	9	60.9 ± 81.4 (2.55)	5	26.6 ± 36.6 (1.51)	9	13.8 ± 44.5 (1.53)	0.395^*^	0.268^†^
At 4.5 years	5	43.6 ± 89.8 (2.00)	2	−9.0 ± 12.7 (0.57)	8	8.4 ± 55.4 (1.30)	0.470^*^	0.559^†^
At 5 years	5	44.8 ± 86.9 (2.25)	4	11.0 ± 27.2 (1.16)	5	−12.6 ± 35.8 (0.92)	0.483^*^	0.333^†^

Values are showed with mean ± SD (fold increase).

The units of mean and SD are marked with ×10^9^/L.

*Student’s t-test between eradicated group and sustained infection group.

^†^ANOVA between the eradicated group, sustained infection group, and no infection group.

SD; standard deviation.

The percentages of patients achieving complete response in each patient group over the study period are described at Supplement Table 2. The CR rates in the eradicated group, sustained infection group, and no infection group were 38.5%, 40.0%, 16.0% (p < 0.001) at 2 months, 36.8%, 16.7%, and 9.5% (p < 0.005) at 6 months, respectively.

### The relationship between the neutrophil-lymphocyte count ratio and *H. pylori* infection status

The baseline NLCR between the eradicated group and the sustained infection group did not show a difference statistically (Table 1). Furthermore, no significant differences in the change of NLCR over time were observed (Supplement Table 1 and Supplement Fig.).

### The relationship between sustained response and eradication success

The eradicated group consisted of 26 patients, of which 25 were analysed for sustained response (SR) achievement and CR duration according to their platelet response at 2 months after eradication. One patient lost follow-up after 2 months of eradication. The results showed that 10 patients showed a sustained response, which was defined as continued CR over six months from the 2-month follow up after the initial eradication (Table 4). Moreover, 7 of the 9 CR patients had their first CR 2 months following eradication and then continued the CR, which was defined as a SR. Among the 25 patients in the eradicated group, 2 patients achieved the response (R) at 2 months following the eradication, 1 patient, non-SR, was CR at the 6^th^ month evaluation and remained above 100 K thereafter. Furthermore, 2 out of the 14 NR patients reached their first CR at 6 months and then showed SR. Patients who reached CR at 2 months following eradication showed SR in 77.8% of the cases. By contrast, in patients who did not achieve CR or R (no response; NR) by the 2-month time point, only 14.3% achieved SR (*P* = 0.010). The mean CR duration for patients with SR in the CR, R, and NR groups at 2 months following eradication were 2.84 ± 1.84 yr [0.5yr–6.0 yr] vs 2.9 yr [2.9yr–2.9 yr] vs 3.3 ± 0.28 yr [3.1yr–3.5 yr], respectively (*P* = 0.946). In the sustained responders, although not statistically significant, older respondents, which comprised a higher proportion of women, had a higher baseline PLT, and lower CRP and ESR. When the 25 patients in the eradicated group were followed, 7 out of 9 CR patients continued to have CR from 2 months following eradication, 1 of 2 patients in the R group reached CR at 6 months and then continued CR. In the eradicated group, 2 out of 14 patients who had no R at 2 months reached CR at 6 months, and R continued thereafter. Differences found for the duration of CR were analysed according to their *H. pylori* infection status in the CR group at 2 months following eradication. A total of 14 patients were analysed, and the CR duration was longer in the eradicated group than in the sustained group; however, no statistical differences were found (2.1 ± 2.0 yr vs 0.9 ± 1.1 yr, *P* = 0.268). Moreover, analysis of 10 of the SR patients revealed that two of the eradicated (splenectomy, additional immunosuppressant) and 1 sustained infection (splenectomy) patients required additional intervention following 2 months of eradication and CR duration increased slightly in the eradicated group than in the sustained infection group; however, this difference was not statistically significant (2.50 ± 1.96 yr vs 1.07 ± 1.19 yr, *P* = 0.169).

**Table 4:** Baseline characteristics between the sustained responder and non-sustained responder in eradicated group.

	Sustained response		Non-sustained response		*P*-value
	N	Mean ± SD	N	Mean ± SD	
Age (year)	10	50.4 ± 9.0	15	54.5 ± 14.3	0.434
Sex (male)	3	37.5%	5	62.5%	0.741
Platelet, ×10^3^/dL	10	61.6 ± 24.6	15	43.2 ± 21.1	0.058
MPV, fL	9	11.5 ± 2.9	12	10.5 ± 1.3	0.287
WBC, ×10^3^/dL	10	8.2 ± 2.2	15	7.7 ± 3.1	0.727
Neutrophil, ×10^3^/dL	10	5.6 ± 2.1	15	4.7 ± 2.3	0.344
Lymphocyte, ×10^3^/dL	10	2.1 ± 0.9	15	2.4 ± 1.2	0.536
Monocyte, ×10^3^/dL	10	0.4 ± 0.2	15	0.4 ± 0.2	0.859
Eosinophil, ×10^3^/dL	10	0.1 ± 0.1	15	0.3 ± 0.5	0.309
Basophil, ×10^3^/dL	10	0.0 ± 0.0	15	0.0 ± 0.0	0.518
Hemoglobin, g/dL	10	13.4 ± 1.8	15	12.3 ± 3.2	0.318
Reticulocyte, g/dL	5	76.3 ± 22.1	9	69.4 ± 32.7	0.685
NLR	10	3.4 ± 4.2	15	1.8 ± 0.8	0.256
CRP, mg/dL	6	0.1 ± 0.1	7	1.3 ± 2.0	0.148
ESR, mm/hr	4	11.0 ± 6.1	6	33.2 ± 30.8	0.201
Antinuclear antibody positivity	0	0.0%	2	100.0%	0.097
**Treatment response at 2 months**					
Complete response	7	77.8%	2	22.2%	0.010
Response	1	50.0%	1	50.0%	
No response	2	14.3%	12	85.7%	

SD; standard deviation, MPV; Mean platelet volume, WBC; White blood cell, NLR; Neutrophil lymphocyte ratio, CRP; C-reactive protein, ESR; erythrocyte sedimentation rate.

## Discussion

In this study, successful eradication of *H. pylori* infected ITP patients tended to increase the platelet count, and sustained *H. pylori* infection, due to a failed eradication attempt, showed poor response to platelet recovery. While eradication was successful in this study, the overall response including CR and R was 46.2% at 2 months following eradication, comparable to 50.3% of the overall response in a meta-analysis with *H. pylori* eradication and platelet recovery^12^. Even if drugs were given concurrently at the initiation of eradication, the first drug initiated could be reduced or stopped within 2 months of eradication. The time to evaluate the therapeutic effect of *H. pylori* eradication treatment for platelet recovery in ITP patients was not previously evaluated^11^. The time required to evaluate the effects of the platelet response may be at least 2 months.

In the general population, the success rate of *H. pylori* eradication was 75.8% using a standard triple therapy within Korea in 2012^13^. A meta-analysis showed that a longer eradication period resulted in a higher eradication rate^14^. Successful eradication with the first-line triple therapy and the success rate of eradication according to the duration of eradication treatment were similar to those of the non-ITP population. Potential methods for increasing the eradication rate of *H. pylori* in the general population are an extended period of treatment or application of tailored therapies using polymerase chain reaction (PCR) to determine antibiotic resistance. If eradication treatment for *H. pylori* is selected as a new strategy for platelet recovery, then, increasing the eradication rate in ITP patients is critical. If the PCR test is not readily available, 14-days standard triple therapy for eradication may be preferred.

The mean platelet counts in the 26 patients with *H. pylori* infection that were not provided eradication therapy was 51.12 ± 36.01 × 10^9^/L. The physician decided that the platelet count was not severe at presentation, and the initial plan for these patients included follow-up visits of the platelet counts without any medications, eradication therapy would be an option if the platelets decreased. Currently, treatment was indicated when the platelet count was less than 3 × 10^10^/L, or related symptoms were found. This study showed that the platelet count could be recovered and maintained within a normal platelet count range after successful eradication of *H. pylori*. Therefore, it was possible to try eradication therapy for *H. pylori* without serious side effects for platelet recovery rather than waiting for the platelets to drop to mild or moderate thrombocytopenia.

Neutrophil lymphocyte count ratio (NLR) is a marker of a systemic inflammatory response and has been reported to be associated with a degree of systemic inflammation or the prognosis of cancer treatment^15,16^. Thus, elevated NLR has been associated with disease activity in autoimmune diseases such as rheumatoid arthritis and systemic lupus erythematosus. However, opposite results have been reported in Behcet’s disease and psoriasis^17^. The significance of the NLR is also controversial. ITP is an autoimmune disease; however, there is limited data regarding ITP and associated inflammatory markers.

Delta platelet count was not significantly different during the early stages but tended to increase. Although this study was a retrospective study, it did not show sufficient statistical significance due to the limitation in the number of patients tracked over a long period. However, it seems that the tendency to increase the platelet count in the eradicated group was maintained when it was based on the baseline platelet count.

In this study, the eradicated group had a tendency of reduced NLR compared to the baseline, but there was no statistical significance. Infection with *H. pylori* has been reported to have higher NLR than patients without this bacterial infection, and this elevated NLR is normalized following the successful eradication of *H. pylori*^18^, which might be associated with the mechanism of systemic inflammation improvement. Further investigations on the effects of HP infection on NLR and platelet recovery are thus warranted.

In a retrospective observational study in Japan, 55% of the 207 patients with ITP who successfully eradicated *H. pylori* retained a partial R after 12 months^19^. In a study by Suzuki *et al*., 25 out of the 36 ITP patients were infected with *H. pylori*, and observed that the platelet levels were elevated in 46.2% of the patients with successful eradication^20^. While in a study by Scandellari in Italy, 16 patients with helicobacter-positive ITP were treated by eradication of *H. pylori*, and found that the increase in platelet count was maintained for 6 months at 43%, and had a CagA antibody positive rate of 83%^21^. Conversely, in Spain, Jarque *et al*. Confirmed that 40 out of 56 ITP patients were infected with *H. pylori* and observed only a platelet increase^22^. In United States, Michel *et al*. Confirmed *H. pylori* infection in 22% of 74 ITP patients, and 14 out of 15 patients who received eradication; however, no platelet recovery was observed^23^. These contradicting reports of the effects of *H. pylori* eradication and platelet response may be due to the prevalence of different characteristics of the *H. pylori* strain between regions, which warrants further studies.

To our knowledge, this is the first study to show the long-term follow-up of platelet counts following eradication of *H. pylori* in ITP in the Korean population. This study showed that successful eradication could affect platelet recovery and the maintenance of the platelet count in patients with ITP.

This study has several limitations. The study was a retrospective single-center design in a country with a high prevalence of *H. pylori* infection. Platelets were not examined at the same time in all patients due to retrospective design. Many patients were excluded from the study since they lacked an assessment of *H. pylori* infection. The virulence of *H. pylori* has not been investigated.

## Conclusion

For the ITP patients with *H. pylori* infection, successful eradication has a role with platelet recovery. In successful eradication with ITP patients.

## Materials and methods

### Study population

Patients with newly diagnosed ITP at Seoul National University Hospital (SNUH) from 2006 to 2016 were retrospectively analysed. The date of diagnosis is defined as the date of the first coding of ITP diagnostic code in electronic medical records of the SNUH. The inclusion criteria were as follows: (1) age 18 years or older, and (2) diagnosis of ITP according to the American Society of Hematology criteria based on an initial platelet count <1 × 10^11^/L^1,2^. The exclusion criteria were as follows: (1) age less than 18 years; (2) thrombocytopenia was related to autoimmune disorders, drugs, a family history consistent with inherited thrombocytopenia, human immunodeficiency virus infection, hepatitis, gestational thrombocytopenia or pseudothrombocytopenia; (3) previous history of *H. pylori* eradication; (4) history of medication with proton pump inhibitors (PPI), H2 antagonists, or antibiotics in the previous 4 weeks; (5) no assessment of *H. pylori* infection; (6) no assessment for the results of *H. pylori* eradication; and (7) other active malignancy.

The patients were classified into three groups, according to *H. pylori*, infection status depicted in Fig. 1. Of the 138 patients, 75 were infected, and 63 were not infected with *H. pylori*. Patients who were diagnosed as *H. pylori* infection and reached negative conversion after the eradication therapy were assigned as the ‘eradicated group’; those diagnosed as *H. pylori* infection but failed eradication or did not performed eradication were reclassified as ‘sustained infection group’ and those without *H. pylori* infection at the initial evaluation were assigned as ‘no infection group’. Detailed analyses were conducted for each of the 61 patients, who were either *H. pylori*-positive and without concomitant or additional therapy until 2 months following eradication, or *H. pylori*-negative and received no treatment for 2 months from the time of diagnosis (Fig. 1).

### Diagnosis of *H. pylori* and eradication therapy

The subjects were assessed for infection with *H. pylori* via one of the following methods: anti-helicobacter antibody (immunoglobulin G), ^13^C-urea breath test (UBT), rapid urease test (CLOtest, HALYARD, Zaventem, Belgium) or Wright Giemsa stain. If at least one of the results was positive, the patient was diagnosed with *H. pylori* infection. Patients diagnosed with *H. pylori* received standard triple therapy (standard dose of PPI twice a day, amoxicillin 1,000 mg twice a day, and clarithromycin 500 mg twice a day) for 7–21 days to eradicate this infection. The results of eradication therapy were assessed by using a UBT, 4 weeks post eradication therapy. If positive UBT results following treatment with the first-line treatment was found treatment was considered a failure and patient was given second-line treatment. Second-line treatment involved bismuth quadruple therapy (standard dose of PPI twice a day, bismuth 120 mg four times a day, metronidazole 500 mg three times a day, and tetracycline 500 mg four times a day for 14 days). The results for the second-line treatment were tested with UBT again and patients with a negative result were placed in the Eradication group, while patients with a positive result were assigned to the Sustained infection group. Patients without *H. pylori* infection were in the No infection group and received no medication.

### Assessment of response with treatment

The changes in platelet count were assessed by treatment response. The treatment response was assessed according to the criteria proposed by the ITP International Working Group guidelines^11^. Complete remission (CR) was determined to have occurred if the platelet count increased to at least 1 × 10^11^/L. R was defined as an increase in platelets to a minimum of 3 × 10^10^/L and at least a two-fold increase from the baseline count combined with the absence of bleeding. No response (NR) was defined as a platelet count that remained below 3 × 10^10^/L, or less than a two-fold increase in the baseline platelet count or bleeding. In this study, the baseline platelet count was defined as the platelet count just before starting the eradication therapy in the eradicated group and sustained infection group, or at the time of diagnosis in the no infection group. The responses were specified with information regarding the concomitant administration of the investigated agent. Patients who maintained a CR for more than six months were classified as ‘sustained responders.’

### Statistical analysis

All statistical analysis was performed using Predictive Analytics Software for Windows version 25.0 (SPSS Inc., IBM, Chicago, IL, USA). Following eradication therapy, assessment of the associations between increased platelet count, age, sex, initial platelet count, mean platelet volume (MPV), white blood cell (WBC) count with differential counts, haemoglobin, reticulocyte count, CRP (C-reactive protein), and ESR (erythrocyte sedimentation rate) were evaluated. Student t-test and analysis of the variance test and were used to evaluate continuous variables, while Pearson’s chi-square test and the Fisher exact test were used to analyse the categorical variables. *P*-values < 0.05 were defined as statistically significant.

### Ethical standards

All procedures followed were in accordance with the ethical standards of the Responsible Committee on Human Experimentation and with the Helsinki Declaration of 1964 and later versions. This study was approved by the Institutional Review Board of SNUH (IRB No. H-1905-068-1033). All patient data were anonymized and de-identified prior to analysis, and thus the requirement for patient consent was waived.

## Supplementary information

Supplementary Tables.

Supplementary Figure S1.
