# Author Correction: *Helicobacter pylori* eradication affects platelet count recovery in immune thrombocytopenia

**DOI:** 10.1038/s41598-020-74316-1

**Published:** 2020-10-20

**Authors:** Ayoung Lee, Junshik Hong, Hyunsoo Chung, Youngil Koh, Soo-Jeong Cho, Ja Min Byun, Sang Gyun Kim, Inho Kim

**Affiliations:** 1 Division of Gastroenterology, Department of Internal Medicine and Liver Research Institute, Seoul National University College of Medicine, Seoul, Republic of Korea; 2 Department of Internal Medicine, Ewha Womans University College of Medicine, Seoul, Republic of Korea; 3 Division of Hematology and Medical Oncology, Department of Internal Medicine, Seoul National University Hospital, Cancer Research Institute, Seoul National University College of Medicine, Seoul, Republic of Korea

Correction to: *Scientific Reports* 10.1038/s41598-020-66460-5, published online 10 June 2020

The original version of this Article omitted an affiliation for Ayoung Lee. The correct affiliations for Ayoung Lee are listed below:

Division of Gastroenterology, Department of Internal Medicine and Liver Research Institute, Seoul National University College of Medicine, Seoul, Republic of Korea.

Department of Internal Medicine, Ewha Womans University College of Medicine, Seoul, Republic of Korea.

This has now been corrected in the HTML and PDF versions of this Article, and in the accompanying Supplementary Material.

