# Supplementary material for: Helicobacter pylori eradication affects platelet count recovery in immune thrombocytopenia
Source: Sci Rep. 2020 Jun 10;10:9370. doi: 10.1038/s41598-020-66460-5 (PMC7287131; doi:10.1038/s41598-020-66460-5)
Supplement: Supplementary file 2 — Supplementary Figure S1. [file 41598_2020_66460_MOESM2_ESM.docx]

***Helicobacter pylori* eradication affects platelet count recovery in immune thrombocytopenia**

*Ayoung Lee^1^, *Junshik Hong^2^, Hyunsoo Chung^1^, Youngil Koh^2^, Soo-Jeong Cho^1^, Ja Min Byun^2^, Sang Gyun Kim^1^, Inho Kim^2^

^1^ Division of Gastroenterology, Department of Internal Medicine and Liver Research Institute, Seoul National University College of Medicine, Seoul, Republic of Korea. ^2^ Division of Hematology and Medical Oncology, Department of Internal Medicine, Seoul National University Hospital, Cancer Research Institute, Seoul National University College of Medicine. * Ayoung Lee and Junshik Hong contributed equally to this manuscript

Corresponding Author: Hyunsoo Chung, M.D. E-mail: hschungmd@gmail.com Co-corresponding Author: Inho Kim, M.D. E-mail: ihkimmd@snu.ac.kr

**Supplement Table 1**. The relationship between the success rate of eradication and the duration of treatment

|  |  | The duration of first line treatment | | *P*-value* |
| --- | --- | --- | --- | --- |
|  |  | < 14 days | ≥ 14 days |  |
| Result of eradication | Success | 26 (66.7%) | 9 (90.0%) | 0.244 |
|  | Fail | 13 (33.3%) | 1 (10.0%) |  |
|  |  |  |  |  |
| * Fisher's exact test |  |  |  |  |

**Supplement table 2. Comparison of complete remission rate for the 5-year follow-up among groups with H. pylori infection status**

|  | Eradicated group | Sustained infection group | No infection group |
| --- | --- | --- | --- |
| At 2 months | 10/26 (38.5) | 4/10 (40.0) | 4/25 (16.0) |
| At 6 months | 7/19 (36.8) | 1/6 (16.7) | 2/21 (9.5) |
| At 1 years | 6/17 (35.3) | 0/7 (0.0) | 3/20 (15.0) |
| At 1.5 years | 6/16 (37.5) | 0/3 (0.0) | 3/16 (18.8) |
| At 2 years | 4/16 (25.0) | 0/7 (0.0) | 3/14 (21.4) |
| At 2.5 years | 5/15 (33.3) | 1/4 (25.0) | 3/13 (23.1) |
| At 3 years | 4/13 (30.8) | 0/6 (0.0) | 0/8 (0.0) |
| At 3.5 years | 3/6 (40.0) | 0/3 (0.0) | 1/9 (11.1) |
| At 4 years | 4/9 (44.4) | 2/5 (40.0) | 1/9 (11.1) |
| At 4.5 years | 2/5 (40.0) | 0/2 (0.0) | 1/8 (12.5) |
| At 5 years | 2/5 (40.0) | 0/4 (0.0) | 0/5 (0.0) |

Numbers represent complete remission patients in each group / total number of patients analysed at each time point (percentage of complete remission patients).

**Supplemental Table 3.** The comparison of neutrophil lymphocyte count ratio between the eradicated group and the sustained infection group

|  | Eradicated group | | Sustained infection group | | *P*-value* |
| --- | --- | --- | --- | --- | --- |
|  | N | Mean ± SD | N | Mean ± SD |  |
| Baseline | 26 | 2.75 ± 3.19 | 10 | 1.37 ± 0.48 | 0.184 |
| At 2 months | 26 | 1.50 ± 0.55 | 10 | 1.82 ± 0.84 | 0.284 |
| At 6 months | 19 | 1.84 ± 1.04 | 6 | 1.63 ± 0.99 | 0.665 |
| At 1 years | 17 | 1.51 ± 0.73 | 7 | 1.48 ± 0.58 | 0.917 |
| At 1.5 years | 16 | 1.68 ± 0.66 | 3 | 1.94 ± 0.68 | 0.547 |
| At 2 years | 16 | 1.92 ± 0.99 | 7 | 1.25 ± 0.56 | 0.116 |
| At 2.5 years | 15 | 1.66 ± 0.74 | 4 | 1.72 ± 0.59 | 0.888 |
| At 3 years | 13 | 1.71 ± 0.65 | 6 | 2.11 ± 1.35 | 0.515 |
| At 3.5 years | 6 | 1.67 ± 0.24 | 3 | 1.79 ± 1.24 | 0.824 |
| At 4 years | 9 | 1.61 ± 1.07 | 5 | 1.59 ± 0.48 | 0.975 |
| At 4.5 years | 5 | 1.56 ± 0.66 | 2 | 1.37 ± 0.45 | 0.722 |
| At 5 years | 5 | 1.81 ± 1.18 | 4 | 2.36 ± 1.29 | 0.526 |

The units of mean and SD are marked with ×10^9^/L

SD, standard deviation

* Student's t-test
